# Markov modeling of performance deterioration in irradiated resistive plate chambers

**DOI:** 10.1140/epjc/s10052-025-15105-w

**Published:** 2025-12-04

**Authors:** Dario Stocco, Michele Pulver, Christian M. Franck

**Affiliations:** Institute for Power Systems and High Voltage Technology, ETH Zurich, 8092 Zurich, Switzerland

## Abstract

This work presents a piecewise deterministic Markov process model for describing performance deterioration in resistive plate chambers (RPC) under uniform background irradiation. The approach accommodates high irradiation rates and arbitrary charge spectra. When applied to the one-dimensional single-cell model of a three-layer single-gap RPC, it shows agreement with state-of-the-art Monte Carlo simulations, which has not been achieved so far. The Markov process model is further applied to the single-cell model with polarizable resistive layers and the two-dimensional counterpart. Moreover, we show that incorporating streamer-like charge events allows the model to reproduce experimental data on efficiency curves under irradiation. These large charge events explain the experimentally observed reduction of the efficiency plateau at high irradiation rates. Extending this insight to eco-friendly gas mixtures reveals that suppressing streamers in general is crucial for achieving high-rate capability.

## Introduction

Finding environmentally friendly gas mixtures for use in resistive plate chambers (RPC) is essential for sustainable operation in future high-radiation environments such as the high-luminosity large hadron collider (LHC) [1]. A performance metric in this context is the rate capability, which quantifies how well an RPC with the installed gas mixture maintains its performance. It is well established that performance decreases with higher background irradiation. For example, efficiency is reduced, and time resolution worsens [2]. Eco-friendly gas mixture candidates based on the eco-friendly molecule HFO1234ze(E) suffer a stronger reduction of the efficiency plateau ($\sim$ 2 %/(100 Hz/cm$^2$)) than the current installed standard mixture ($\sim$ 0.7 %/(100 Hz/cm$^2$)) [3]. This efficiency reduction presents a serious limitation for their application in the high-luminosity LHC. Theoretical models to explain these effects are limited. They are either based on Monte Carlo (MC) sampling methods (for details, see Section 2.1 in [4]) or on Campbell’s theorem to calculate the mean voltage drop across the gas gap and, from that, the performance drop [5–7]. Both approaches assume that charge deposition $Q_{\textrm{dep}}$ from background-induced discharges (e.g., electron avalanches and streamers) leads to a temporary local voltage drop across the gas gap. In general, the gap voltage $V_{\textrm{gap}}$, defined as the voltage across the gas gap at a given point on the RPC area $(x, y) \in A_{\textrm{RPC}}$ and time *t* evolves as

1 $$\begin{aligned} V_{\textrm{gap}}(x, y, t)  &   = V_{\textrm{app}} + \sum _{i} Q_{\textrm{dep}}\left( V_{\textrm{gap}}(x_i, y_i, t_i)\right) \nonumber \\  &   \quad \cdot \Delta V'(x - x_i, y - y_i, t - t_i), \end{aligned}$$

where $V_{\textrm{app}}$ is the externally applied voltage to the RPC, $Q_{\textrm{dep}}\left( V_{\textrm{gap}}(x_i, y_i, t_i)\right)$ is the deposited charge sampled at the given gap voltage from the voltage dependent charge distribution $\rho _{Q_{\textrm{dep}}}(q| V_{\textrm{gap}})$. The times $t_i$ are the time-stamps of background events with mean rate $\Phi A_{\text {RPC}}$, where $\Phi$ is the flux of the background radiation. $\Delta V'(x,y,t)$ is the localized voltage drop per unit charge.

In our recent work, we demonstrated that Campbell’s theorem allows for estimating the stationary spectrum of the abundant gap voltages $\rho _{V_\textrm{gap}}(V)$ due to fluctuations from Eq. (1). Ultimately and generally, this gap voltage spectrum enables the estimation of the performance drop under radiation and is thus one of the main ingredients in modeling irradiated RPCs. For example, the efficiency curve under irradiation can be expressed as the product of the abundant gap voltages and the efficiency curve at no rate condition [4],

2 $$\begin{aligned} \varepsilon (V_{\textrm{app}}, \Phi ) = \int \textrm{d}V \ \rho _{V_{\textrm{gap}}}(V) \varepsilon (V, \Phi = 0). \end{aligned}$$

However, our study also showed that applying Campbell’s theorem together with a marked Poisson process (mPP), which describes the dynamical charge deposition from the background radiation, is only valid up to a certain background rate (see Figure 4 in [4]). The mPP marks each background event with a charge based on the average gap voltage spectrum rather than the instantaneous time-dependent gap voltage. At high rates, the correlation between events becomes stronger, and the average spectrum is too broad to determine the charge of the next event.

In this publication, we extend the theoretical model beyond this rate limit. Furthermore, the proposed extension allows estimating the rate capability with arbitrary charge spectrum shapes within the single-cell model suggested by Abbrescia [5]. For two-dimensional models, we propose using the cell matching criterion instead of solving the associated equations to get the gap voltage spectrum. We then demonstrate that streamer-like discharges cause the efficiency plateau to drop under irradiation by explicitly applying the single-cell model to measured data from a three-layer single-gap RPC. Eco-friendly gas mixtures typically quench streamers less effectively and are more prone to this drop, reducing their rate-capability.

## Piecewise deterministic Markov process

As noted in the introduction, the approach using the mPP based on the Campbell theorem [6, 7] to compute the gap voltage spectrum according to Eq. (1) ignores the strong correlation between subsequent events and tries to capture it via the relation $\rho _Q = \int \textrm{d}V \ \rho _{Q_{dep}} \rho _{V_{gap}}$ [4]. As a result, this formalism fails to describe the case of high irradiation and high deposited charges. The correct approach is to include the correlation between events. In some cases, when the RPC geometry, material properties, and discharge mechanism are not too complex, the process can be described as a piecewise deterministic Markov process (PDMP) [8, 9]. Section 3 provides three examples, where PDMP is applicable.

The PDMP is a continuous-time Markov process consisting of three ingredients to determine the distribution of a random vector $\textbf{X}_t$ at time *t*. In the use case of irradiated resistive plate chambers, the random vector $\textbf{X}_t$ is typically the gap voltage $V_{\textrm{gap}}$. The first ingredient is the deterministic evolution of $\textbf{X}_t$ between subsequent background events

3 $$\begin{aligned} \frac{\textrm{d} \textbf{X}^{(i)}_t}{\textrm{d} t} = \Phi _i(\textbf{X}_t). \end{aligned}$$

In our application, the evolution corresponds to the recovery of the gap voltage dictated by $\Delta V'$ due to the removal of charge by conduction through the resistive layers. The second ingredient is the event rate, which for one-dimensional models with constant hit rate is given by $\lambda = \Phi A$ (*A* is either the cell size $A_{\textrm{cell}}$ in case of one-dimensional models or the area spanned by the RPC $A_{\textrm{RPC}}$) and does not depend on $\textbf{X}_t$. Finally, the last and most important ingredient is the transition distribution at each event at time $t^*$, given by $q(\cdot | \textbf{X}_{t^*_-})$. That describes the random jump from state $\textbf{X}_{t^*_-}$ right before the transition to the state $\textbf{X}_{t^*}$, such that,

4 $$\begin{aligned} \textbf{X}_{t^*} \sim q(\cdot | \textbf{X}_{t^*_-}). \end{aligned}$$

dictated by $q(\cdot | \cdot )$. The density *q* captures the correlation between subsequent events, while the jump corresponds to the instantaneous voltage drop caused by the charge deposition from a background event. The time dependence of the distribution $\textbf{X}_t \sim \rho _t(\textbf{X})$ is then given by the equation,

5 $$\begin{aligned} \partial _t \rho _t(\textbf{X})  &   = - \sum _{i=1}^d \partial _{X^{(i)}} \left( \Phi _i(\textbf{X}) \rho _t(\textbf{X})\right) \nonumber \\  &   \quad + \lambda \int \textrm{d}\mathbf {X'} \rho _t(\mathbf {X'}) q(\textbf{X} | \mathbf {X'}) - \lambda \rho _t(\textbf{X}), \end{aligned}$$

also known as the Fokker–Planck- or forward Kolmogorov equation. Setting $\partial _t \rho _t(\textbf{X}) = 0$ gives the stationary distribution $\rho _{t\rightarrow \infty }(\textbf{X})$ and corresponds to the distribution of abundant voltages across the gas gap $\rho _{V_{\textrm{gap}}}(V)$.

## Results

We present the application of the PDMP, more specifically the stationary Fokker–Planck equation (5), to the single-cell model of a three-layer single-gap RPC. In one case, the resistive electrodes have constant permittivity; in the other, their permittivity follows Debye relaxation. We also apply the equations to a two-dimensional extension of a two-layer single-gap RPC with constant-permittivity electrodes, accounting for spatial variations of the gap voltage. If possible, we provide expressions for the mean and variance of the gap voltage. However, this depends on the charge deposition spectrum and may not always be feasible. For the single-cell model with constant permittivity, we show how to solve the equations to obtain the full gap voltage spectrum.

### Single-cell model with constant permittivity

The single-cell model is described in more detail in [4]. Briefly, it divides the RPC into cells of size $A_{\textrm{cell}}$, each treated as the equivalent RC-circuit for constant-permittivity electrodes. To solve the Fokker–Planck equation (5) for this model, we only need to consider the gap voltage evolution $\textbf{X} = V_{\textrm{gap}} = V$. It evolves in time as

6 $$\begin{aligned} \Phi _1(V) = \frac{V_{\textrm{app}} - V}{\tau _3}, \end{aligned}$$

with the three-layer time constant $\tau _3 = \rho (\epsilon _r + \frac{2 d_r}{d_g}\epsilon _0)$ ($d_r$, $\epsilon _r$, and $\rho$ the thickness, permittivity, and resistivity of the two resistive layers, $d_g$ the thickness of the gas gap, and $\epsilon _0$ the vacuum permittivity). Jumps from *V* to $V'$ occur with the transition probability density,

7 $$\begin{aligned} q(V'|V) = \frac{1}{\Delta V'_{0}}\rho _{Q_{\textrm{dep}}}\left( \frac{V - V'}{\Delta V'_{0}}| V \right) , \end{aligned}$$

with the charge normalized voltage drop $\Delta V'_{0} = 2d_r/A_{\textrm{cell}} \tau _3 \sigma$ ($\sigma = \rho ^{-1}$ the conductivity) at the instant of the event ($t = t^{*}$).

The following delay differential equation (DDE) determines the stationary gap voltage distribution based on these ingredients and the stationary Fokker–Planck equation,

8 $$\begin{aligned} \partial _V \rho _{V_{\textrm{gap}}}(V)  &   = \frac{1}{V_{\textrm{app}}-V}\left\{ \left( 1 - \Phi A_{\textrm{cell}}\tau _3\right) \rho _{V_{\textrm{gap}}}(V) + \Phi A_{\textrm{cell}}\tau _3\right. \nonumber \\  &   \quad \left. \int _{V}^{V_{\textrm{app}}} \frac{\textrm{d}V'}{\Delta V'_0} \rho _{Q_{\textrm{dep}}}\left( \frac{V' - V}{\Delta V'_0}|V'\right) \rho _{V_{\textrm{gap}}}(V')\right\} , \end{aligned}$$

for any charge deposition spectrum given at no irradiation condition $\rho _{Q_{\textrm{dep}}}(q| V)$. The integration of $V'$ starts at *V* up to $V_{\textrm{app}}$ because outside these bounds, either the charge distribution is zero or the voltage distribution vanishes.

#### Mean and variance at equilibrium – delta-like charge spectrum with linear mean

Considering the delta-like charge deposition spectrum at no irradiation

9 $$\begin{aligned} \rho _{Q_{\textrm{dep}}}(q|V) = \delta (q - \alpha (V-V_{\textrm{th}})) \end{aligned}$$

the mean and variance of the gap voltage calculated from the stationary Fokker–Planck equations are given by

10 $$\begin{aligned} \langle V_{\textrm{gap}}\rangle = V_{\textrm{th}} + \frac{V_{\textrm{app}} - V_{\textrm{th}}}{1 + \Phi \frac{2d_r}{\sigma } \alpha }, \end{aligned}$$

what was already correctly reproduced with the marked Poisson process approach [4], and

11 $$\begin{aligned}  &   \textrm{Var}(V_{\textrm{gap}})(V_{\textrm{app}}, \Phi ) \nonumber \\  &   \quad = \frac{\Phi A_{\textrm{cell}}\tau _3 (\alpha \Delta V'_0)^2}{2 + \Phi A_{\textrm{cell}}\tau _3 \alpha \Delta V'_0(2-\alpha \Delta V'_0)} \left( \langle V_{\textrm{gap}}\rangle - V_{\textrm{th}}\right) ^2. \nonumber \\ \end{aligned}$$

**Fig. 1:**
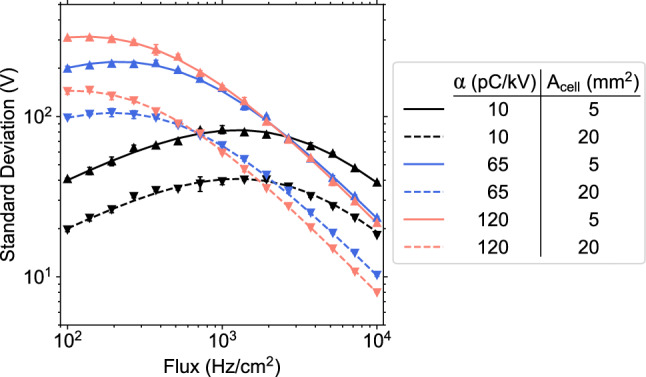
The standard deviation of the gap voltage given the single-cell model and a linear delta-like charge deposition spectrum. Solid lines represent the analytical expression derived in this work, and scatter points represent the Monte Carlo simulation, including statistical uncertainty. The colors indicate different cell sizes and charge-to-voltage ratios $\alpha$

Figure 1 shows the standard deviation computed by MC simulations and Eq. (11) for a broad range of $\alpha$ and $A_{\textrm{cell}}$ and typical values for trigger RPCs. Both approaches yield the same values within the uncertainties.

If the spectrum is not delta-like but satisfies $\textrm{Var}(Q_{\textrm{dep}}) / \langle$ $Q_{\textrm{dep}} \rangle ^2 = \mathrm {const.} =:\kappa ^2$, then the variance is given by,

12 $$\begin{aligned}  &   \textrm{Var}(V_{\textrm{gap}})(V_{\textrm{app}}, \Phi ) \nonumber \\  &   \quad = \frac{\Phi A_{\textrm{cell}}\tau _3 (1 + \kappa ^2)(\alpha \Delta V'_0)^2}{2 + \Phi A_{\textrm{cell}}\tau _3 \alpha \Delta V'_0(2-(1 + \kappa ^2)\alpha \Delta V'_0)} \nonumber \\  &   \qquad \cdot \left( \langle V_{\textrm{gap}}\rangle - V_{\textrm{th}}\right) ^2. \end{aligned}$$

The validity of the marked Poisson process model in [4], given by an upper limit on the rate, can be evaluated by comparing the analytic expressions of the variances (equation (26) from [4]) and introducing a safety parameter $0< \eta < 1$,

13 $$\begin{aligned} \Phi _{\textrm{max}}^{(\textrm{mPP})} = \frac{1}{2}\frac{\eta }{\frac{\alpha d_g}{\sigma } (1 + \eta \frac{\alpha d_g}{\sigma \tau _3 A_{\textrm{cell}}} )} \sim \frac{1}{\alpha }, \end{aligned}$$

which works for the 2D models as well, considering the correct cell size matching criterion. The reciprocal dependency on the charge-to-voltage coefficient $\alpha$ is identified from it.

#### Solving the DDE – delta-like charge spectrum with linear mean

Rewriting the DDE (8) using the charge spectrum (9) by introducing the variables $x:= - V_{\textrm{gap}}$ and $y(x):= \rho _{\textrm{Vgap}}(-V)$ leads to

14 $$\begin{aligned} \partial _x y(x) = \frac{1}{x - x_0}\left\{ (\Phi A_{\textrm{cell}} \tau - 1)y(x) -\frac{\Phi A_{\textrm{cell}} \tau }{1 - \alpha \Delta V_0'}y(x - \chi (x))\right\} , \nonumber \\ \end{aligned}$$

with the delay function

15 $$\begin{aligned} \chi (x) = \frac{\alpha \Delta V_0'}{1 - \alpha \Delta V_0'}(x_1-x), \end{aligned}$$

where $x_0 = -V_{\textrm{app}}$ and $x_1=-V_{\textrm{th}}$. Thus the breakpoints ($x^{(n)} - \chi (x^{(n)}) = x^{(n-1)}$) of this equation are at $x^{(n)}=(1-\alpha \Delta V_0')^n x_0 + (1 - (1-\alpha \Delta V_0')^n)x_1$. They divide the interval $(x_0, x_1 = \mathrm {lim_{n\rightarrow \infty }}x^{(n)} )$ in increasing order $x^{(n-1)}< x^{(n)} < x^{(n+1)}$. Thus the DDE can be solved iteratively since for $x \in (x^{(n)}, x^{(n+1)})$, the delay $x- \chi (x)$ reaches the preceding interval, $(x^{(n-1)}, x^{(n)})$. For the first interval considering $y(x<x_0) = 0$ the DDE can be solved analytically,

16 $$\begin{aligned} y^{(1)}(x) = \mathbbm {c}_1 (x - x_0)^{\phi A_{\textrm{cell}} \tau - 1}. \end{aligned}$$

**Fig. 2:**
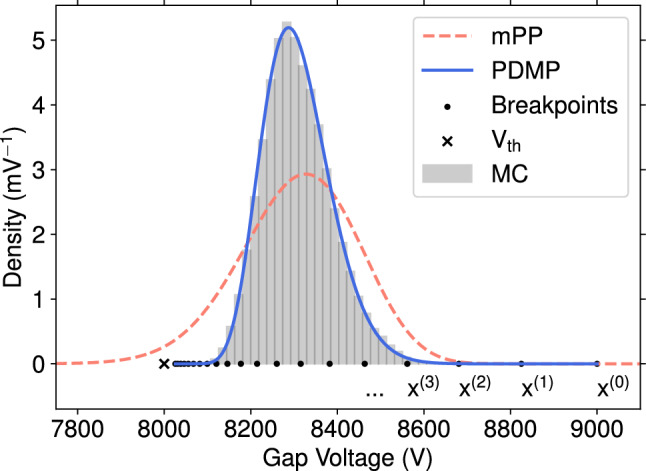
Solution of the stationary Fokker–Planck equation with a linear delta-like charge deposition spectrum in blue (PDMP) compared to Monte Carlo in gray (MC) and the semi-analytical model in red (mPP). The breakpoints are labeled as $x^{(i)}$

The constant $\mathbbm {c}_1$ is chosen such that *y*(*x*) is a probability density function, i.e., the area under the curve is 1. With the solution on the first interval, the solution of the following intervals can be solved with the initial condition given by $y^{(n)}(x^{(n-1)}) = y^{(n-1)}(x^{(n-1)})$. Solving this can be done, for example, with scipy.integrate.solve_ivp. Figure 2 shows an example of the gap voltage spectrum calculated by solving the DDE (labeled PDMP), the solution from the marked Poisson process [4] (labeled mPP), and compared to MC calculations. The rate was set to 1.6 kHz $\hbox {cm}^{-2}$ and the charge-to-voltage coefficient to $\alpha = {70}\,\hbox {pC\,\,kV}^{-1}$.

For $\Phi A_{\textrm{cell}} \tau > 1$, the solution does not have any poles; however, for $\Phi A_{\textrm{cell}} \tau < 1$ a pole at $V = V_{\textrm{app}}$ arises due to the negative exponent in Eq. (16). This pole propagates through the iteration and causes numerical issues. A numerical approximate solution is possible if one evaluates the initial value of *y*(*x*) not at $x=x_0$ but at a slight shift $x=x_0 + \epsilon$, for $0<\epsilon<< 1$. Comparison with MC simulations indicates that shifting by $\epsilon$ provides accurate results.

#### Solving the DDE – arbitrary charge spectrum

By introducing the variables $x = V_{\textrm{app}} - V$ and $y(x) = \rho _{V_\textrm{gap}}(V_{\textrm{app}} - V)$, the DDE equation (8) is rewritten in the form

17 $$\begin{aligned}  &   \partial _x y(x) = \frac{1}{x}\left\{ (\Phi A_{\textrm{cell}}\tau - 1)y(x)\right. \nonumber \\    &   \left. \quad {-} \Phi A_{\textrm{cell}} \tau \int _0^x\frac{\textrm{d}x'}{\Delta V_0'} \rho _{Q_{\textrm{dep}}} \left( \frac{x - x'}{\Delta V_0'} |V_{\textrm{app}} -x'\right) y(x') \right\} .\nonumber \\ \end{aligned}$$

and can be solved by an adaptation of the explicit Euler method, which includes the history of the so far calculated solution. A solution should exist as long as the charge deposition spectrum is smooth enough. Another caveat is that the initial value for $\Phi A\tau > 1$ is $y(0) = 0$, which leads numerically to the solution $y(x) = 0$. Similar to the linear delta case, for small *x* the solution is again given by expression (16). Thus, one can simply set as initial value any $y(\epsilon ) = y_0 > 0$ for any small $\epsilon >0$. The final solution must be normalized to one, removing the *free* choice made for $y_0$. Solving the DDE can be up to $\sim$100 times faster than the MC time-evolution approach. A straightforward calculation of the mean voltage drop is not possible if the average charge deposition is nonlinear, because the term $\rho _{\textrm{gap}}(V) \cdot \langle Q_{\textrm{dep}} \rangle (V)$ appears in the calculation and depends on the shape of the gap voltage spectrum.

### Single-cell model with Debye-like relaxation

Assuming that the frequency-dependent permittivity of the resistive electrode behaves as the Debye relaxation model [17],

18 $$\begin{aligned} \epsilon (\omega ) = \epsilon _{\infty } + \frac{\epsilon _r - \epsilon _{\infty }}{1 + i \omega \tau _1}, \end{aligned}$$

with $\epsilon _r$ the DC permittivity, $\epsilon _{\infty }$ the saturated permittivity at high frequency, and $\tau _1$ the relaxation time. It must not necessarily be that the physical process involved in the Debye model is due to dipole or similar relaxations. The derived results can be used as long as the measured frequency-dependent permittivity shows a similar shape. Then the deterministic gap voltage evolution of a cell can be described by two dependent variables $\textbf{X} = (\Delta V_1, \Delta V_2)^T$, i.e., $V_{\textrm{gap}}(t) =V_{\textrm{app}} + \Delta V_1(t) + \Delta V_2(t)$. They evolve as

19 $$\begin{aligned} {\varvec{\Phi }}(\Delta V_1, \Delta V_2) = \begin{pmatrix} -\frac{1}{\tau _{0,1}} &  0 \\ 0 &  -\frac{1}{\tau _{0,2}} \end{pmatrix} \begin{pmatrix} \Delta V_1 \\ \Delta V_2 \end{pmatrix} \end{aligned}$$

with the two characteristic times $\tau _{0,i}$, which are given by the solutions of the quadratic equation

20 $$\begin{aligned} 0 = \tau ^2 - \left( \tau _1 + \tau _3^{(r)}\right) \tau + \tau _3^{(\infty )}\tau _1, \end{aligned}$$

where $\tau _3^{(i)}$ is the time constant of the three-layer RPC with permittivity $\epsilon _r =\epsilon _{i}$. The transition density couples the two voltage drops,

21 $$\begin{aligned}&\nonumber q(\Delta V'_1,\Delta V'_2|\Delta V_1,\Delta V_2) = \frac{1}{\Delta V_{0,1}'}\rho _{Q_{\textrm{dep}}} \left( \frac{\Delta V_1-\Delta V'_1}{\Delta V_{0,1}'}| V_{\textrm{app}}+\Delta V_1+\Delta V_2\right) \nonumber \\&\quad \cdot \delta \left( \Delta V_2'-\Delta V_2-\frac{\Delta V_{0,2}'}{\Delta V_{0,1}'}(\Delta V_1' - \Delta V_1)\right) \end{aligned}$$

where the immediate charge normalized voltage drop $\Delta V'_{0,i}$ is given by,

22 $$\begin{aligned} \Delta V_{0,1}' =&\Delta V'_{\infty } \frac{1-\frac{\tau _{0,1}}{\tau _1}}{1-\frac{\tau _{0,1}}{\tau _{0,2}}}, \nonumber \\ \quad \Delta V_{0,2}' =&\Delta V'_{\infty } \frac{1-\frac{\tau _{0,2}}{\tau _1}}{1-\frac{\tau _{0,2}}{\tau _{0,1}}}, \\ \quad \Delta V'_{\infty } =&\frac{2d_r }{A_{\textrm{cell}}\sigma \tau _3^{(\infty )}}. \nonumber \end{aligned}$$

#### Mean and variance at equilibrium – delta-like charge spectrum with linear mean

Considering again the charge spectrum given by Eq. (9). Applying the stationary Fokker–Planck equations yields the same mean voltage drop like in the case with constant permittivity given by Eq. (10) but the variance differs,

23 $$\begin{aligned} \textrm{Var}(V_{\textrm{gap}}) =&\frac{\hat{\tau }^2\left( \alpha \Delta V'_{\infty }\right) ^2 A_{\textrm{cell}}\Phi (\langle V_{\textrm{gap}}\rangle -V_{\textrm{th}})^2}{ \hat{\tau }^2 \left( 2\frac{\tau _{0,1}+\tau _{0,2}}{\tau _{0,1}\tau _{0,2}} + \alpha \Delta V'_{\infty } A_{\textrm{cell}}\Phi \left( 2 - \alpha \Delta V'_{\infty }\right) \right) - \left( 2(\tau _{0,1}+\tau _{0,2}) + 2\alpha \Delta V'_{\infty } A_{\textrm{cell}}\Phi \tau _{0,1}\tau _{0,2}\right) } \\ \nonumber \text {with} \quad \hat{\tau }^2 :=&\tau _1^2 +\tau _{0,1}\tau _{0,2}\left( 1 + \alpha \Delta V'_{\infty }A_{\textrm{cell}}\Phi \tau _1\right) . \end{aligned}$$

**Fig. 3:**
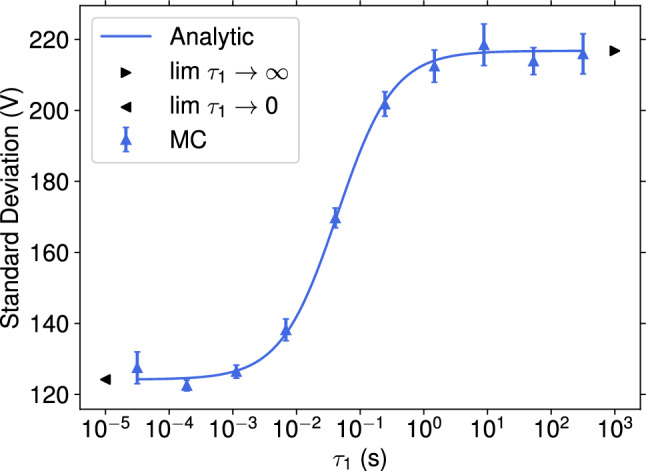
Comparison of the standard deviation of the gap voltage for different relaxation times $\tau _1$. The blue solid line is the analytic expression, and the blue scatter points are the Monte Carlo (MC) prediction, including statistical uncertainty. For the MC simulation, the solution of Eq. (19) is used to model the localized voltage drop per unit charge $\Delta V'$. The black scatter points, the two relaxation time limits, are calculated by the analytic expression for constant permittivity, which shows that both expressions coincide in the limit

The effect of the relaxation time constant $\tau _1$ on the gap voltage variation is conceptually illustrated in Fig. 3. The Figure shows a resonance approximately given by the RPC geometry-dependent time constant $\tau _3^{(r)}$ based on the DC permittivity $\epsilon _r$. Materials with time constants $\tau _1$ much larger than the resonance time can be considered by a constant permittivity of $\epsilon (\omega ) = \epsilon _{\infty }$ and vice versa, i.e., a much smaller time constant with $\epsilon (\omega ) = \epsilon _{r}$. Regarding rate capability, small gap voltage variations lead to less loss of efficiency, and from that, one can conclude that materials with small time constants (resonance at large frequencies) and or large $\epsilon _r$ are preferable. It is expected to find resonances with relaxation times much smaller than $\tau _3^{(r)}$, and we don’t have to worry about the polarization of the electrodes [10].

### Two-dimensional model: bulk resistivity

As in [4], the two-dimensional model describes the transport of charges originating from a charged disk with radius *R* across the resistive layers. The problem is cylindrical symmetric. Compared to the single cell model, the charge transport occurs not only along the axial direction but also in the radial direction. For the two-layer single gap RPC with bulk resistivity $\rho$, where both layers have the same thickness *d*, a useful simplification arises. The voltage drop of a single event separates into independent functions of time and radial distance $\Delta V'(t, r) = \textrm{e}^{-t/\tau _2}\Delta V'(r)$. When we then consider that the spatial (*x*, *y*) hits are uniformly distributed (the time is still a Poisson process with rate $\lambda = \Phi A_{\textrm{RPC}}$), then the radial hit distances can be considered as a random variable with density,

24 $$\begin{aligned} \rho _{\textrm{hit}}(r) = \frac{2\pi r}{A_{\textrm{RPC}}}, \quad r \in [0, r^*], \end{aligned}$$

with fixed upper radial bound $\pi (r^*)^2:=A_{\textrm{RPC}}$. The deterministic evolution of the gap voltage at the center is modeled identically to the single-cell case. However, the transition density of the voltage is given by,

25 $$\begin{aligned} V' \sim \int \frac{\textrm{d}(\pi r^2)}{A_{\textrm{RPC}}} \frac{\rho _{Q_{\textrm{dep}}}\left( \frac{V - V'}{\Delta V'(r)}| V \right) }{\Delta V'(r)} = q(V'|V). \end{aligned}$$

These definitions produce the same mean value as the single-cell model. The variance is similar to the single-cell case by replacing $A_{\textrm{cell}} \Delta V_0'^{i=1,2} \rightarrow \int \textrm{d}(\pi r^2) \Delta V'(r)^{i=1,2}$. For the $i=1$ case, we already showed that,

26 $$\begin{aligned} \int \textrm{d}(\pi r^2) \Delta V'(r) = \frac{d}{(\epsilon _r + \epsilon _0)}. \end{aligned}$$

Thus, by matching the variance with the constant permittivity single-cell model (in this case: the three-layer single-gap RPC with $d_r \rightarrow d_r/2$), the relation between the cell size and the discharge radius is given by,

27 $$\begin{aligned} A_{\textrm{cell}} := \frac{d^2}{2 \sigma ^2 \tau _2} \frac{1}{\langle \left( \Delta V'_{\textrm{2D}}\right) ^2\rangle (d, R)}, \end{aligned}$$

with

28 $$\begin{aligned} \langle \left( \Delta V'_{\textrm{2D}}\right) ^2\rangle (d, R) = \int _0^{r*} \textrm{d}(\pi r^2) \Delta V'(r). \end{aligned}$$

The same expression has already been derived when modeling the irradiation process with a marked Poisson process [4].

**Fig. 4:**
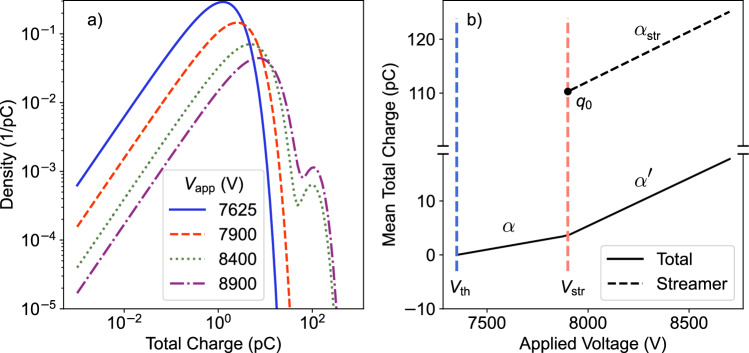
**a** Charge spectrum including large charge events and **b** the associated mean charge at no irradiation condition

**Table 1:** Optimal parameters estimated of the charge deposition spectrum from gammas for single-gap thin-RPCs filled with the standard mixture at the GIF++ facility

$\rho$	$A_{\textrm{cell}}$	$\alpha$	$\alpha '$	$\Phi _{\text {ABS = 22, d = {3} m}}$	$\Phi _{\text {ABS = 22, d = {6} m}}$
8.62e10 $\times 10^{10} \Omega$ cm	37.2 $\hbox {mm}^{2}$	5.4 pC $\hbox {kV}^{-1}$	30.0 pC $\hbox {kV}^{-1}$	373 Hz $\hbox {cm}^{-2}$	105 Hz $\hbox {cm}^{-2}$

**Fig. 5:**
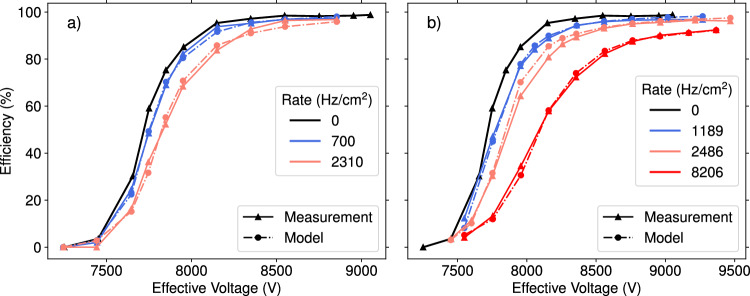
Efficiency curves at different distances to the gamma source **a** 6 m, **b** 3 m. The modeled data is shown as circles, the measured data as triangles. The effective hit rate (converted gammas) used in the simulation is given in the legend

### Comment on the mean current

To calculate the mean current, consider $\textbf{X} = (V=V_{\textrm{gap}}, I)$ where *I* is the current per cell and the coupled density $\rho (V, I)$. The solution to the Fokker–Planck equation for the gap voltage stays the same when considering the marginal density $\rho _{V_{\textrm{gap}}}(V) = \int \textrm{d}I \ \rho (V, I)$ and the deterministic jump in current $\delta (\frac{V - V'}{\Delta V'_0} - \frac{I' - I}{\Delta I'_0})$ ($I'_0$ is the charge normalized current at $t=0$) multiplied to the jump probability $q(\cdot |\cdot )$. Applying the Fokker–Planck equation to the marginal density $\rho _{I}(I) = \int \textrm{d}V \ \rho (V, I)$ leads to the following relation between the mean current and gap voltage,

29 $$\begin{aligned} \frac{2d_r \rho }{A_{\textrm{cell}}} \langle I \rangle = V_{\textrm{app}} - \langle V_{\textrm{gap}}\rangle . \end{aligned}$$

Considering that the total current is $N = A_{\textrm{RPC}}/A_{\textrm{cell}}$ times larger, the DC model is recognized with $R= 2d_r \rho /A_{\textrm{RPC}}$, where the factor of two accounts for two equally resistive layers. For the two-dimensional model, the approach will be the same, by replacing the constant voltage drop with the radially dependent voltage drop.

## Application

The model describing the gap voltage distribution, based on Eq. (17) together with the deterioration Eq. (2), is, as an example, applied to reproduce the efficiency curves under irradiation of a thin RPC from the ECOGas@GIF++ collaboration filled with the standard mixture [11]. They used a three-layer single-gap RPC, where both resistive layers and gas gap have a thickness of 1.6 mm. The background radiation consists of a Cesium-137 source emitting gammas with an energy of 662 keV. There is limited information on the charge spectrum of gammas for this type of RPC. For 511 keV photons and multi-gap RPCs, the avalanche mode charge spectrum was measured in [12, 13], showing a $\Gamma$-distribution-like shape. Measurements from [14] show that the average gamma charge from a Cobalt-60 source increases linearly until streamer contributions begin, after which it rises more rapidly.

In this work, we place special emphasis on large charge events, such as those resulting from discharges due to streamers. Inspired by the above-mentioned experimental results, we consider a piecewise linear average charge, with slope $\alpha$ [pC/kV] between $V_{\textrm{th}}$ and the streamer inception voltage $V_{\textrm{str}}$, and slope $\alpha '$ [pC/kV] above the streamer inception voltage. For the streamers, it is considered that they deposit an average charge linearly increasing with the voltage $q_0 + \alpha _{\textrm{str}} [\text {pC/kV}] (V - V_{\textrm{str}}), \ V > V_{\textrm{str}}$. The right plot of Fig. 4 illustrates the mean total charge and mean streamer charge of the underlying charge distribution. Based on the mean charge, we assume that two $\Gamma$ distributions describe the avalanche and streamer spectra, as shown on the left plot in Fig. 4. The distributions are parametrized by a shaping parameter $\theta$,

30 $$\begin{aligned} \rho _{Q_{\textrm{dep}}}(q| V) \propto q^{\theta } \textrm{exp}\left( -\frac{(\theta + 1)q}{\langle Q_{\textrm{dep}}\rangle (V)} \right) . \end{aligned}$$

For the avalanche spectrum, we set $\theta$ = 1, and for the streamer spectrum, we set it to 5.

We fix the turn-on voltage $V_{\textrm{th}}$ to 7350 V, guided by the shape of the measured efficiency curves. Further, we fix the streamer inception voltage $V_{\textrm{str}}$ to 8000 V, based on results from avalanche-streamer separation with muons. The value of the streamer inception voltage dictates the rate-dependent efficiency decrease below the knee, and the chosen value appears reasonable. The irradiation rate $\Phi$, the rate of converted gammas or the hit-rate, is scaled according to the ratios of the attenuation filter values (ABS). This scaling leaves one irradiation rate as a free parameter per experimental configuration, i.e., for each distance to the Cesium source (3 m and 6 m). Based on empirical testing, the charge spectrum model shows only slight sensitivity to the streamer-associated parameters $q_0$ and $\alpha _{\text {str}}$, which are therefore fixed to 110 pC and 50 pC $\hbox {kV}^{-1}$, respectively. Fitting the remaining free parameters of the charge spectrum, the resistivity of the electrodes, the one irradiation rate per configuration, and the cell size to the efficiency curves under irradiation yields the optimal parameters shown in Table 1. Interestingly, the cell size is relatively large with 37.2 $\hbox {mm}^{2}$, which corresponds to a discharge area of around 19.1 $\hbox {mm}^{2}$ in the two-dimensional model according to Eq. (27). It is clear that, in contrast to muons hitting the RPC at normal incidence in the GIF++ beam experiments, high-energy primary electrons released from gamma-ray interaction (dominantly Compton scattering) further ionize the gas molecules along paths that are not necessarily perpendicular to the detector surface. The resulting spread of primary electrons increases the effective area of influence.

The modeled deteriorated efficiency curves with the optimal parameters are shown in Fig. 5 alongside the measurements. These results show that a reduction in the efficiency plateau occurs due to the large charge events caused by streamers (parameter $\alpha '$). Increasing the streamer charges, i.e., $\alpha '$, further decreases the efficiency plateau even more. Conversely, reducing the streamer charges lifts the efficiency plateau.

When the data is presented as a function of the average gap voltage $\langle V_{\textrm{gap}}\rangle =V_{\textrm{app}} - R \langle I \rangle$, all the efficiency curves overlap. This overlap can be attributed to the fact that the variance of the gas gap voltage scales inversely with the cell size. Consequently, for the large estimated cell size, the efficiency weighted by the gap voltage spectrum is dominated by the mean gap voltage. However, some authors experimentally observe a different trend. For example, in [15, Fig. 4.44], the DC model correction is too weak and the efficiency curves don’t overlap. In contrast, others observe the opposite, where the efficiency, after DC model correction, increases at fixed mean gap voltage for higher irradiation rates [16, Fig. 4.54 and Fig. 4.55]. The latter could be an indication of highly non-uniform fields perpendicular to the RPC surface, which have not been considered in the formalism so far.

## Conclusion

We present the application of a piecewise deterministic Markov process (PDMP) to describe the irradiation process of resistive plate chambers (RPCs) analytically. This description extends the literature with an analytical model that applies to arbitrary rates and charges. Based on the stationary Fokker–Planck equation, a consequence of the PDMP, and assuming linear average charge deposition, the mean and variance of the gas gap voltage drop are calculated. Results are presented for the single-cell model, with and without polarizable electrodes, and for the two-dimensional model without polarizable electrodes. These expressions match Monte Carlo simulations for high rates and high charges.

Furthermore, we show how the gap voltage probability density for arbitrary charge spectra can be numerically calculated in the case of the single-cell model. We subsequently demonstrate its application to measured efficiencies under gamma irradiation of a three-layer single-thin-gap RPC (1.6 mm). Charge deposition is modeled using a piecewise-linear average charge model that includes large charge events (streamers). This model fits experimentally observed data well and allows us to explain the reduction of the efficiency plateau, which could not be reproduced without the large charge events. Independent of the charge spectrum model, we can conclude that streamer-suppressing gas mixtures possess a higher rate capability. This makes them particularly important when evaluating suitable eco-friendly alternatives for RPCs.

Limitations of the PDMP include its Markov property, the assumption of uniform fields, and the mostly unknown charge spectrum of the background irradiation. If the RPC geometry includes more complicated processes, like surface resistive electrodes, the Markov property cannot be guaranteed. Such cases most likely must be examined with Monte Carlo simulation techniques. We always assume uniform fields perpendicular to the RPC surface, which is strictly speaking wrong. Local surface charges lead to non-uniform field distortions in the direction perpendicular and tangent to the RPC surface. The unknown charge spectrum could be measured, for example, at low rates such that individual events don’t interfere with each other.

## Data Availability

Data will be made available on reasonable request. [Authors’ comment: The datasets generated during and/or analysed during the current study are available from the corresponding author on reasonable request.]

## Code Availability Statement

Code/software will be made available on reasonable request. [Authors’ comment: The code/software generated during and/or analysed during the current study is available from the corresponding author on reasonable request.]
